# RDF graph pair profile dataset for the data linking community

**DOI:** 10.1016/j.dib.2024.111017

**Published:** 2024-10-10

**Authors:** Raphaël Conde Salazar, Clément Jonquet, Danai Symeonidou

**Affiliations:** a MISTEA, University of Montpellier, INRAE & Institut Agro, France, 2, place Pierre Viala, 34060 Montpellier Cedex 2, France; b LIRMM, University of Montpellier & CNRS, France, 161 Rue Ada, 34095 Montpellier cedex 5, France

**Keywords:** RDF graph statistics, RDF graph profiles, Dataset pairing, Ontology matching

## Abstract

As the number of RDF datasets published on the Web grows, it becomes increasingly important to link similar entities across these datasets. We present the “RDF graph pair profiles dataset”, designed to help the data linking community develop tools and carry out evaluation work. This dataset includes profiles of 30 RDF graph pairs, classified according to ontology matching (OM), instance matching (IM) or both (OM + IM). Each profile includes statistical measures and lists of qualitative and quantitative information and descriptive models generated using automated tools. These profiles help in understanding dataset characteristics, facilitating the development, selection and validation of data linking tools. They are particularly useful in machine learning applications where the profiles can serve as input parameters. The dataset includes both the quasi-original RDF graphs and their profiles represented in a specific described format offering a comprehensive resource for researchers and practitioners. The methodology applied to obtain the profiles is also briefly presented.

Available publicly (DOI: 10.57745/K7JDGV) this dataset will facilitate data linking, hence contribute to the integration and enhancement of RDF data published in the Web of data.

Specifications Table

**Table:** 

Subject	Data Science, Data Mining and Statistical Analysis
Specific subject area	This dataset provides RDF graph pair profiles to support data linking. It is useful for tool development and validation as well as machine learning training.
Type of data	- Original RDF graphs are RDF data format serialised in XML syntax available in text (TXT) files); - Profiles are rendered in the form of JSON text files (around 40 per dataset) and two CSV format files.
Data collection	The data collection process involved first: selecting and gathering the original RDF graphs to profile; second: establishing the respective profiles of pairs of RDF graphs. These profiles are composed of statistical criteria as well as quantitative and qualitative lists. They were created automatically by a program developed for this purpose, and saved in JSON format and as tables in CSV format. When RDF graph pairs were used in benchmarking campaigns for data linking tools (in our case, OAEI), the performance of the tools evaluated was recorded along with the profiling results. 30 pairs have been processed and are provided in the dataset. They are divided into three groups to be used according to the type of linking envisaged (23 pairs for Ontology Matching (OM), 2 pairs for Instance Matching (IM) or 5 for both (OM + IM).
Data source location	Institution: INRAE French National Research Institute for Agriculture, Food and the Environment. City/Town/Region: Montpelier–– N; 3°51′17.17″ E
Data accessibility	Repository name: Recherche data gouv [1] Profiles: https://doi.org/10.57745/WKW8MR RDF graphs pairs: https://doi.org/10.57745/5IQMST Instructions for accessing and using these data are also available in the same collection (https://entrepot.recherche.data.gouv.fr/dataverse/datasetpairprofiling) with the dedicated DOI: 10.57745/K7JDGV.

## 1 Value of the Data

• We provide the data linking community with access to profiles of pairs of RDF graphs that can be used in multiple data linking tasks.
• These profiles provide specialists with the means to develop and evaluate methods and tools for linking data.
• This data can also be used with machine learning approaches to train and evaluate models for improving data linking.
• Our dataset is a valuable resource for semantic data linking, the process of linking RDF data from one source to another to consolidate the integration of data and knowledge within RDF knowledge graphs. By linking information from multiple sources to create a coherent overview, it improves the accuracy of analyses, facilitates decision-making based on complete data, and helps identify relationships and trends that are not explicit.

## 2 Background

As the number of RDF datasets on the semantic Web continues to increase, efficiently linking similar entities across these datasets becomes increasingly important. Various data linking approaches include deterministic linking using unique identifiers, probabilistic linkage with non-unique identifiers [[2], [3], [4]], rule-based linking with rules to specify the linking conditions [5,6], knowledge graph integration representing relationships as translations in a embedding space [7], and more recently, machine learning techniques.

Whatever the approach, designers of data linking tools use pairs of semantic datasets that must and can be linked. According to the terminology used by this community within a pair, the graphs are called “source” and “target”, and the set of semantic links between individuals in the two graphs is called “reference”.

In the case of data-centric approaches such as the DACE-DL project [8], which promotes a bottom-up approach to data linking, designers and developers also need a more complete profile of source and target graphs, the results of which can be fed directly into machine learning processes. Although previous works exist for the constitution of dataset profiles [[9], [10], [11]], our work stands out by taking into account pairs of RDF graphs for the data linking community and by providing a larger number of metrics (62 in our case versus 32 for LODStats).

We hope that this dataset will contribute to the collective knowledge of the field, support current and future research in data linking, and serve as an important resource for researchers to create or improve their tools.

## 3 Data Description

Here we describe the dataset structure and how to access it.

**Fig. 1:**
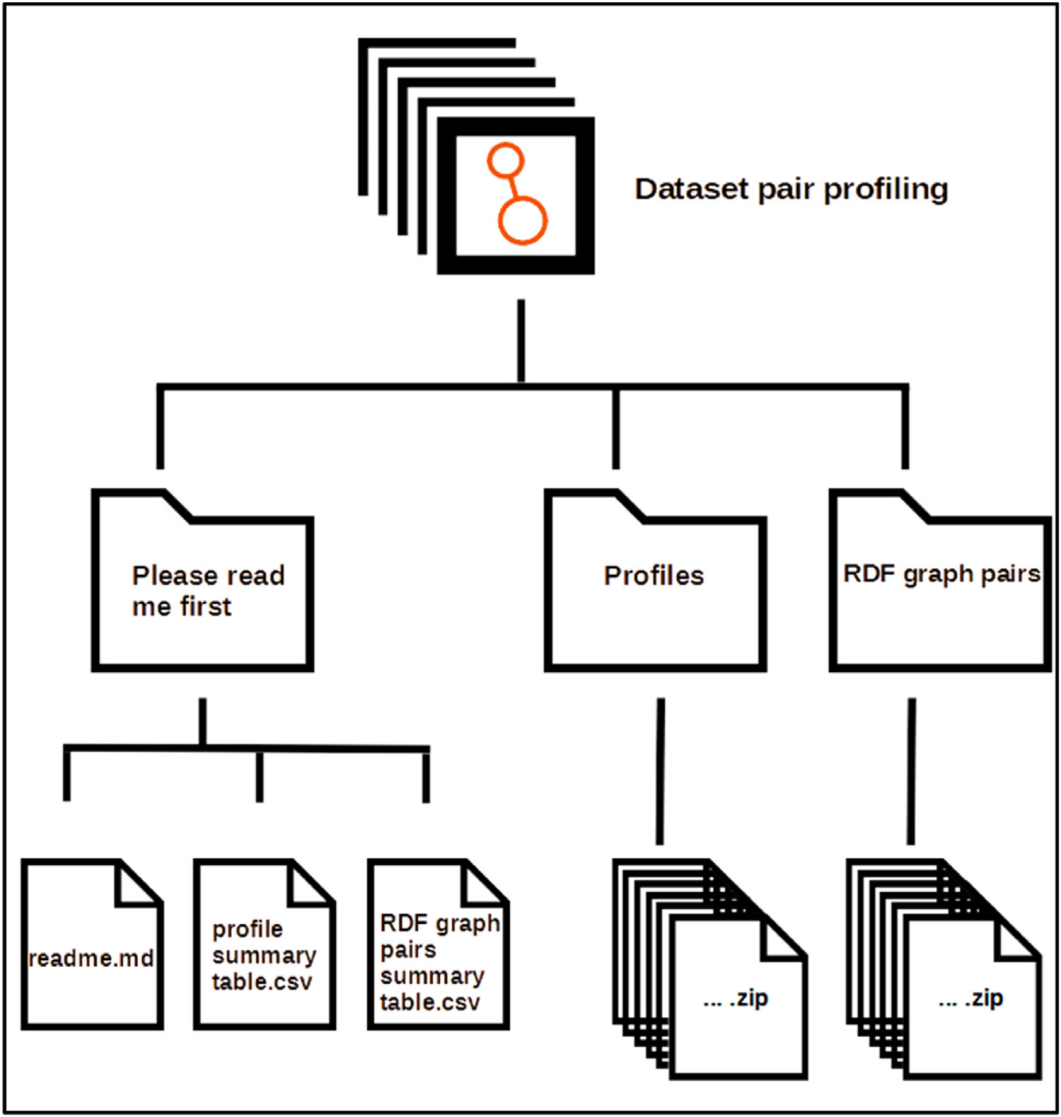
General structure of the dataset.

This dataset is organised in three main folders: “Please read me first”, “Profiles” and “RDF graphs pairs” has is shown in Fig. 1.

Folder and their contents:

• Folder “Please read me first”
  - The files here provide important information for understanding and navigating the “Profiles” and “RDG graphs pairs” folders.
  - readme.md is a file containing general explanations of the dataset.
  - profile summary table.csv: is a tabular file detailing the structure and summaries of the dataset profiles.
  - RDF graph pairs summary table.csv is a tabular file that details the structure and summaries of the RDF graphs peers used for profiling.
• Folder “Profiles”

This directory contains ZIP archive files, each containing profiling results for a pair of RDF graphs [12]. In the context of ontologies and RDF graphs for knowledge representation, the TBox (Terminological Box) describes the conceptual structure of an RDF graph, including classes, properties and hierarchies and the ABox (Assertion Box) contains the concrete instances of these concepts and the relationships between them. Together, they can be used to define and populate an RDF knowledge base. The names of these ZIP archive files are standardised: The “OM-” prefix corresponds to the profiling results of a pair of graphs concerning models (Tbox). This prefix is followed by the year in which the graphs were exploited and then the name of the source graph and the target graph. The “IM-” prefix corresponds to pairs of graphs containing only instances (Abox). The absence of a prefix in front of the year indicates that the RDF graphs used provide both models and instances (Tbox and Abox) (see Fig. 6). Profiles include statistical metrics as well as quantitative and qualitative lists for better understanding the RDF graphs. These ZIP archives contain the files that make up the source and target graph profiles (see Fig. 7).

The profiles generated for the source and target RDG graphs of a pair can be classified into four groups:

• Statistics
• Quantitative and qualitative lists
• Description model
• Linking problem types exposed

### 3.1 The statistics

**Fig. 2:**
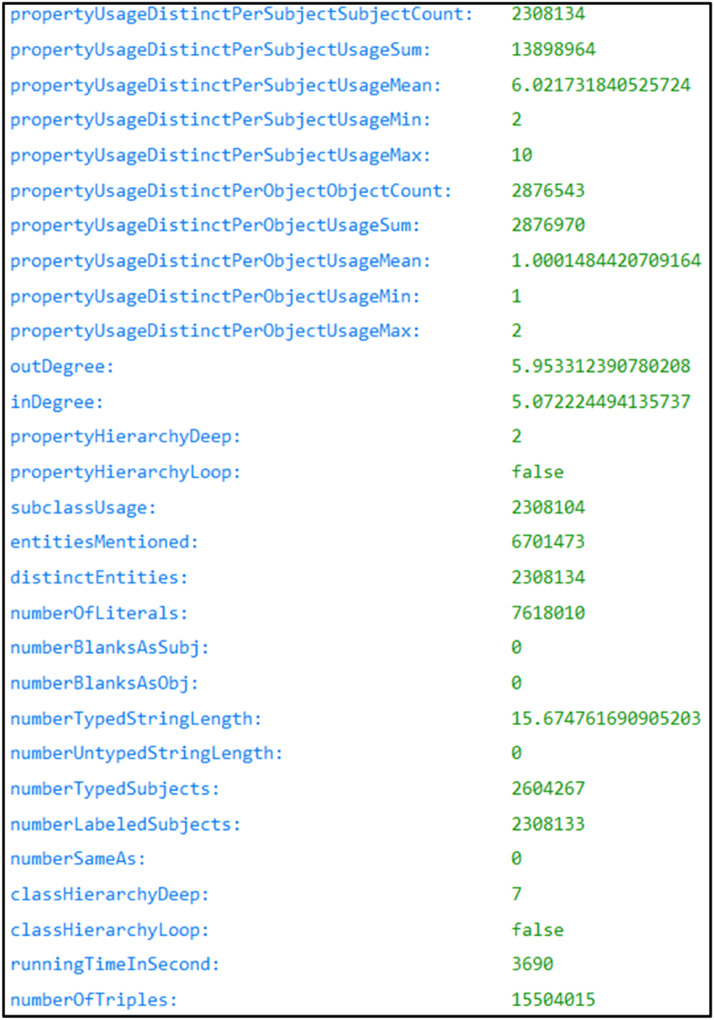
Example of statistics for the RDG graph of the NCBI Taxonomy [13].

A total of 29 statistics have been extracted. They are summarised in the Table 1. The various statistics extracted are presented as key-value pairs in the results.json file (see Fig. 2).

**Table 1:** The 29 metrics extracted by the dataset pair profiling program.

Variable	Type	Description
propertyUsageDistinctPerSubjectSubjectCount	Integer	Number of distinct subjects using specific properties.
propertyUsageDistinctPerSubjectUsageSum	Integer	Total sum of distinct property usages per subject.
propertyUsageDistinctPerSubjectUsageMean	Float	Average number of distinct property usages per subject.
propertyUsageDistinctPerSubjectUsageMin	Integer	Minimum number of distinct property usages by a subject.
propertyUsageDistinctPerSubjectUsageMax	Integer	Maximum number of distinct property usages by a subject.
propertyUsageDistinctPerObjectObjectCount	Integer	Number of distinct objects using specific properties.
propertyUsageDistinctPerObjectUsageSum	Integer	Total sum of distinct property usages per object.
propertyUsageDistinctPerObjectUsageMean	Float	Average number of distinct property usages per object.
propertyUsageDistinctPerObjectUsageMin	Integer	Minimum number of distinct property usages by an object.
propertyUsageDistinctPerObjectUsageMax	Integer	Maximum number of distinct property usages by an object.
outDegree	Float	Average out-degree, i.e., the average number of outgoing links per subject.
inDegree	Float	Average in-degree, i.e., the average number of incoming links per object.
propertyHierarchyDeep	Integer	Depth of the property hierarchy.
propertyHierarchyLoop	Boolean	Indicates if there are loops in the property hierarchy.
subclassUsage	Integer	Number of rdfs:subclassOf property usage.
entitiesMentioned	Integer	Total number of IRIs present in all triplets in the graph.
distinctEntities	Integer	Total number of distinct IRIs present in all triplets in the graph.
numberOfLiterals	Integer	Number of literals present in the graph.
numberBlanksAsSubj	Integer	Number of blank (anonymous) subjects.
numberBlanksAsObj	Integer	Number of blank (anonymous) objects.
numberTypedStringLength	Float	Average length of typed strings.
numberUntypedStringLength	Float	Average length of untyped strings.
numberTypedSubjects	Integer	Number of typed subjects (having a specified type).
numberLabeledSubjects	Integer	Number of labelled subjects (having a label).
numberSameAs	Integer	Number of usages of the “sameAs” property (entity equivalence).
classHierarchyDeep	Integer	Depth of the class hierarchy.
classHierarchyLoop	Boolean	Indicates if there are loops in the class hierarchy.
runningTimeInSecond	Integer	Execution time in seconds for processing the data.
numberOfTriples	Integer	Total number of triples contained in the RDF graph.

### 3.2 The quantitative and qualitative lists

A total of 33 quantitative and qualitative lists were generated. Their names and descriptions are summarised in the Table 2.

**Table 2:** The 33 quantitative and qualitative lists extracted by the Dataset Pair Profiling program.

File Name	Type	Description
listClassAndSubclass.json	Json	Contains data on classes and their subclasses.
listClassDefined.json	Json	Contains data on classes that are explicitly defined.
listClassLanguages.json	Json	Contains data on the languages used in class definitions.
listClassMostUsed.json	Json	Contains data on the most used classes (Usage above the fourth quartile).
listClassNotDefined.json	Json	Contains data on classes that are not explicitly defined.
listClassUsageCount.json	Json	Contains counts of how frequently each class is used.
listCombinationPropertiesClassRelationships.json	Json	Contains data on combinations of properties and class relationships.
listCombinationPropertiesClassRelationshipsClasses.json	Json	Contains data on combinations of properties and their related classes.
listCombinationPropertiesClassRelationshipsPropertiesOfClasses.json	Json	Contains data on properties of classes within combinations of properties and class relationships.
listCombinationPropertiesClassRelationshipsRelationships.json	Json	Contains data on the relationships within combinations of properties and class relationships.
listCombinationPropertiesPerSubject.json	Json	Contains data on combinations of properties per subject.
listCombinationPropertiesPerSubjectCleanedAndReduced.json	Json	Contains cleaned and reduced data on combinations of properties per subject.
listCombinationPropertiesWithNewClass.json	Json	Contains data on combinations of properties including newly identified classes.
listLinks.json	Json	Contains data on links between various entities.(With different domain names)
listMaxPerProperty.json	Json	Contains data on the maximum of the values observed per property.
listMostUsedAnnotationProperty.json	Json	Contains data on the most used annotation properties.
listMostUsedDatatypeProperty.json	Json	Contains data on the most used datatype properties.
listMostUsedObjectProperty.json	Json	Contains data on the most used object properties.
listMostUsedProperty.json	Json	Contains data on the most used properties overall (The first 100).
listMostUsedPropertyDatatypes.json	Json	Contains data on the datatypes of the values of the most used properties.
listMostUsedPropertyType.json	Json	Contains data on the types of the most used properties.(When explicitly indicated)
listMostUsedPropertyUsagePerObject.json	Json	Contains data on the usage of the most used properties per object.
listMostUsedPropertyUsagePerSubject.json	Json	Contains data on the usage of the most used properties per subject.
listMostUsedPropertyWithClassDomain.json	Json	Contains data on the most commonly used properties and their domains.
listMostUsedPropertyWithDatatypeAnd ClassRange.json	Json	Contains data on the most used properties and their datatype and class ranges.
listMostUsedRDFproperty.json	Json	Contains data on the most used RDF properties.
listObjectVocabulary.json	Json	Contains data on the vocabulary used for objects.
listOfDatatypes.json	Json	Contains a list of datatypes used in the dataset.
listOfLanguagesPredicat.json	Json	Contains data on the languages used to describe predicates.
listOfLanguagesPredicatValue.json	Json	Contains data on the language used for (literal) predicate values.
listPerProperty.json	Json	Contains average values per property. (datatype = integer, float, double, decimal.)
listPredicatVocabulary.json	Json	Contains data on the vocabulary used for predicates.
listPropertyAndSubproperty.json	Json	Contains data on properties and their subproperties.
listPropertyUsageCount.json	Json	Contains counts of how frequently each property is used.
listSharedPartObjectVocabulary.json	Json	Contains data on shared parts of the object vocabulary.
listSharedPartSubjectVocabulary.json	Json	Contains data on shared parts of the subject vocabulary.
listSubjectVocabulary.json	Json	Contains data on the vocabulary used for subjects.

These lists provide essential information about classes, properties and relationships in an RDF graph. Moreover, they enable analysis of data structure, usage and interconnections, facilitating model analysis and optimization. Some examples of some are provided below:

1. Classes and Properties:
  • listClassAndSubclass.json: Details the hierarchical relationships between classes.
  • listClassDefined.json: Lists classes defined in the model.
  • listMostUsedObjectProperty.json: Indicates the most frequently used object properties.
2. Usage and Frequency:
  • listClassMostUsed.json: Identifies the most frequently used classes.
  • listPropertyUsageCount.json: Shows how often each property is used.
  • listMostUsedPropertyUsagePerSubject.json: Shows property usage by subject.
3. Vocabulary and Data Types:
  • listObjectVocabulary.json: Provides the vocabulary of objects used.
  • listOfDatatypes.json: Lists the datatypes used.
  • listPredicatVocabulary.json: Provides the vocabulary of predicates.
4. Combinations and Relationships:
  • listCombinationPropertiesPerSubject.json: Presents property combinations for each subject, allowing detailed subject characteristics to be analysed.
  • listCombinationPropertiesClassRelationships.json: Describes class relationships, revealing complex patterns in the data.
  • listLinks.json: Provides a list of links between entities.

These metrics are important for understanding the structure of RDF data, enabling in-depth analysis and targeted optimization. For example, knowing the most frequently used properties can help identify the key attributes of a graph, while vocabulary lists provide an overview of the terminologies used.

**Fig. 3:**
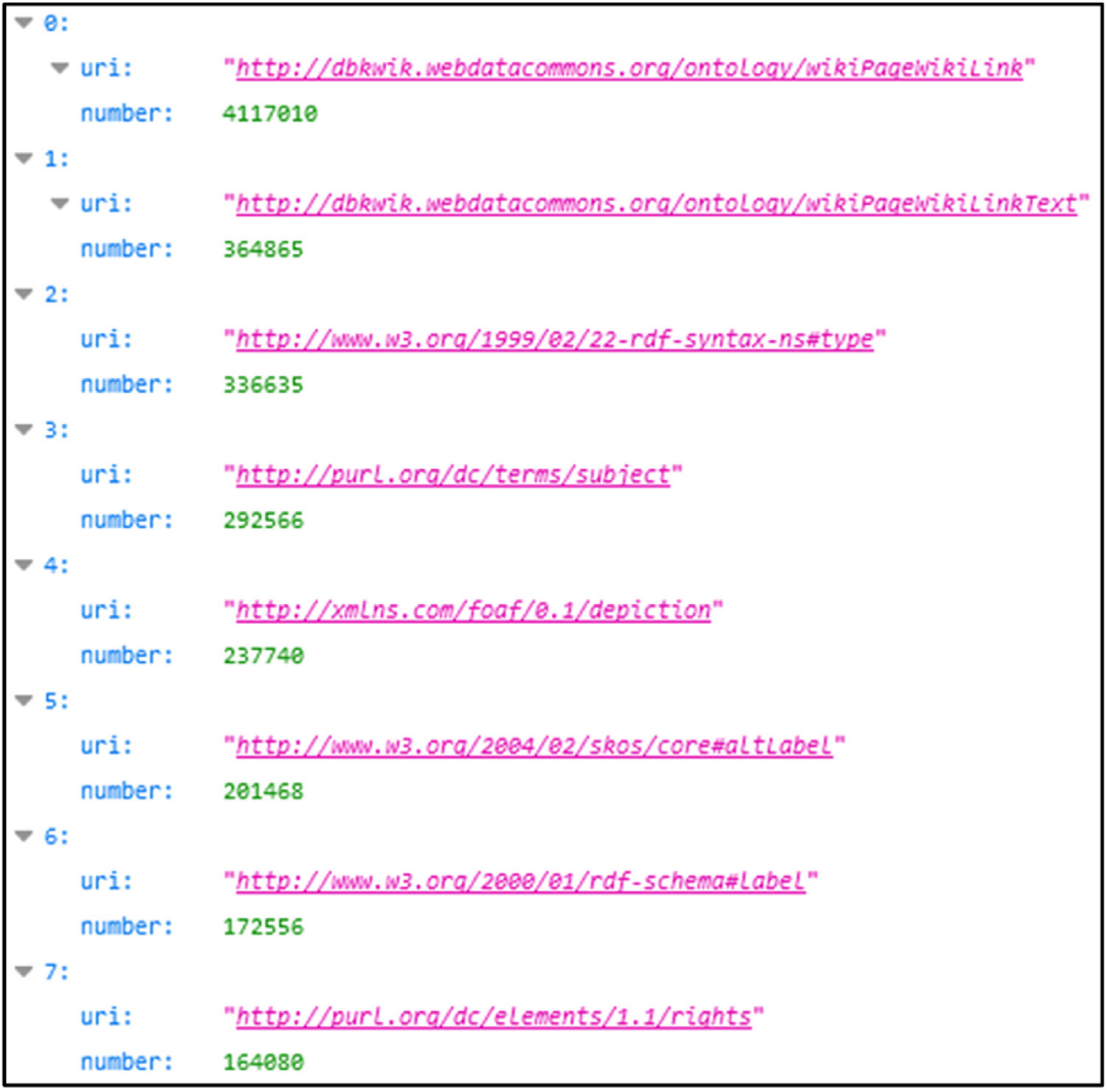
Extract from the listPropertyUsageCount.json list for the RDF graph corresponding to the Starwars ontology [15].

An example of data representation in JSON format for the quantitative listPropertyUsageCount.json is shown in Fig. 3. The “uri” field indicates the URI of the property and the “number” field the number of occurrences of this property within the triplets of the graph.

### 3.3 The description model

**Fig. 4:**
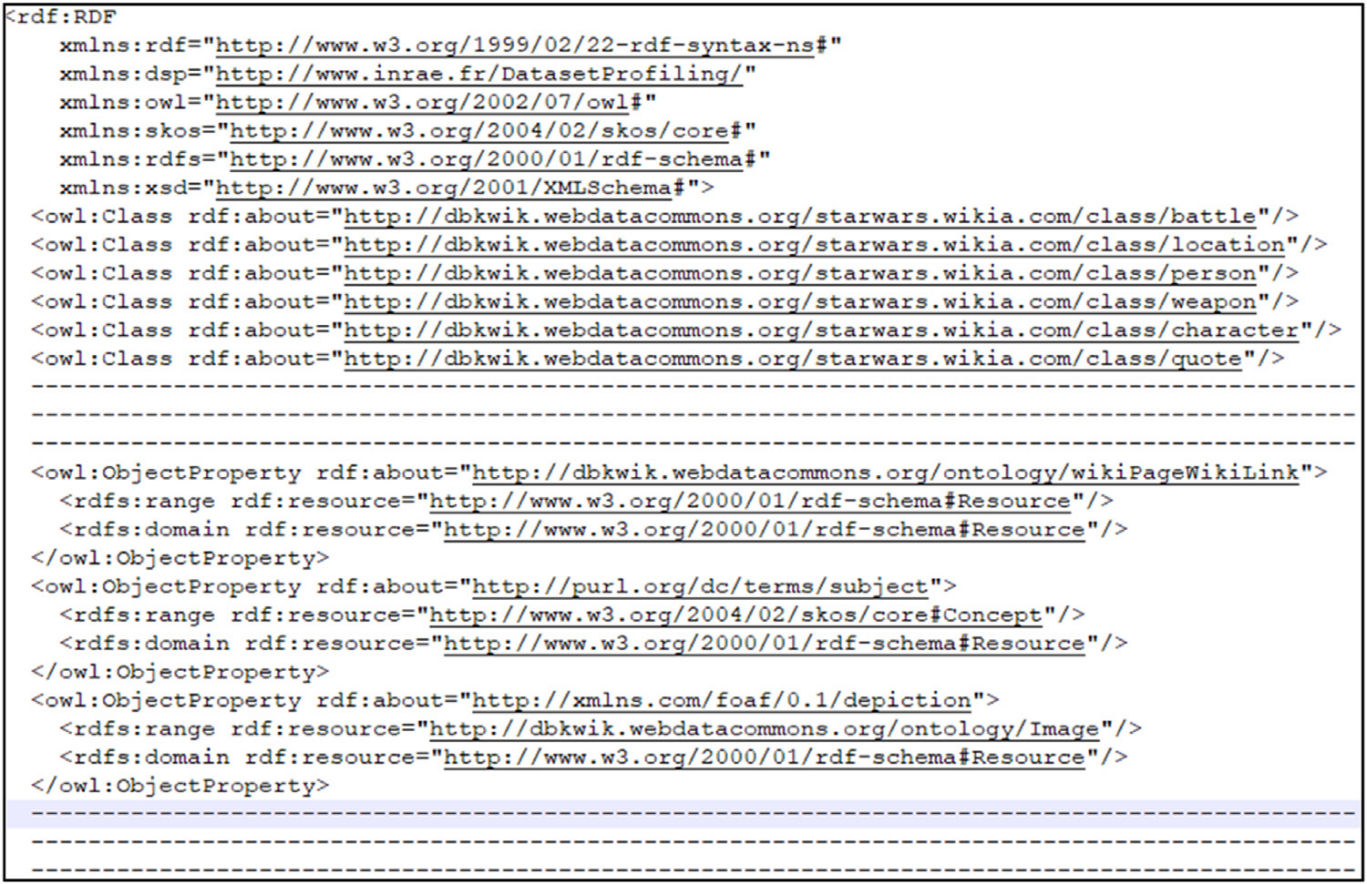
Extract from the descriptionModel.owl file for the RDF graph corresponding to the Starwars ontology [15].

For each graph, we present a descriptive model in the form of an OWL ontology (see Fig. 4) contained in the descriptionModel.owl file (see Fig. 7).

### 3.4 The linking problem types exposed

**Fig. 5:**
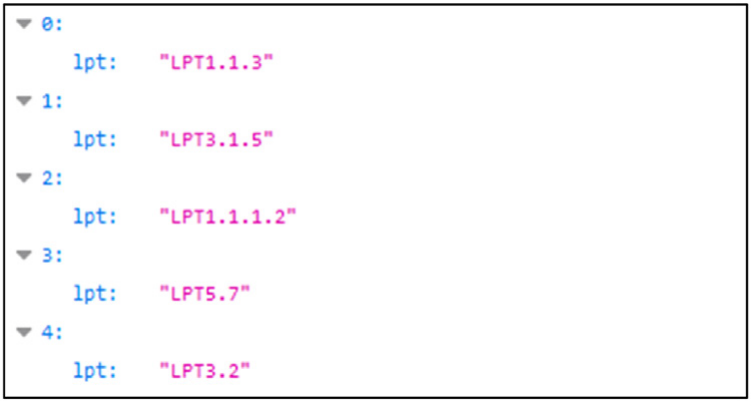
LPTs corresponding to the pair of dataset Taxrefld [13] and NCBI Taxonomy [13].

Linking Problem Types (LPTs) are problems that can be encountered during a semantic data linking process. For each pair of graphs, we present a list of LPTs contained in the resultingLPTs.json file (see Fig. 7). Fig. 5 shows an example of this list for the dataset pair Taxrefld [13] and NCBI Taxonomy [14].

• Folder “RDF graph pairs”

**Fig. 6:**
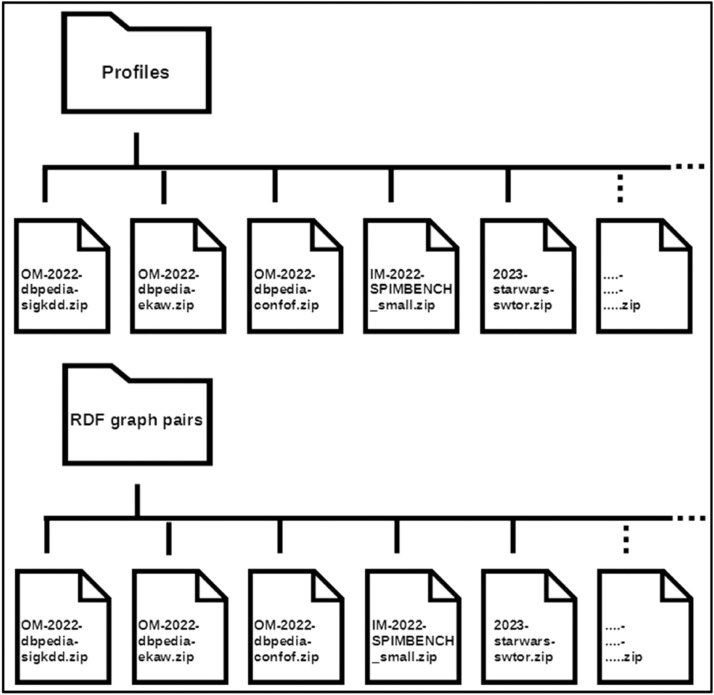
Profiles and Datasets directory structure.

This folder contains ZIP archive files. Each ZIP file contains the pairs of RDF graphs that have been processed. The names of the files in this directory correspond to the names of the files in the Profiles directory, to make it easier to compare and use the data (see Fig. 6). As shown in Fig. 8, each of these archive files contains the pair of graphs used (source.xml and target.xml) and, if available, an alignment reference (reference.xml) between the resources in these two graphs.

## 4 Experimental Design, Materials and Methods

The profiles for each dataset pair were generated by a set of programs called “Dataset Pair Profiling” developed for this purpose.

By establishing profiles of the ``source'' and ``target'' graphs for a pair of graphs, our aim is to facilitate the process of creating semantic links between individuals in these two graphs, as shown in Fig. 9.

The RDF graph pairs processed come from the Ontology Alignment Evaluation Initiative (OAEI) [15] for the years 2022 and 2023, which organises annual benchmarks for designers of ontology alignment and data linking tools. Despite the availability of these RDF graphs on the Web, we nevertheless decided to make them available in our dataset. Indeed, for archiving and reproducibility reasons, it was important to save them, as the original RDF graphs are not hosted in long-term data repositories or identified by PIDs. Plus, some of the RDG graphs required curation for problems related to serialisation or syntax e.g. invalid characters or malformed URIs. The version of the RDF graphs in the dataset are cleaned from these small errors by the “Dataset Pair Profiling” program and can be used right away. We have chosen to use the RDF graph pairs used during the OAEI benchmarking sessions, as they are provided with entity equivalences between each graph of the same pair, validated by community experts. Prior knowledge of these links is essential for the future validation of tools that will use the profiles we have generated for development and validation purposes.

The variables and lists extracted by this program are partly inspired by the work done to develop the LODStats framework [9] (particularly for statistics). For the choice of the content of the qualitative and quantitative lists, we consulted developers of data linking tools, in particular those specialized in machine learning processes. The various variables and lists are described in detail in the “Data description” section.

The various stages of the program are:

- Curation and transfer of the various graph pairs supplied as RDF/XML files to a specialized RDF triplet database (TDB jena).
- Creation of statistics and lists, as well as a description model for each graph in a pair.
- Creation of a list of LPTs.
- Storage of results.

The “Dataset Pair Profiling” program is written in JAVA language with the Jena toolbox specialized in RDF graph processing.

As mentioned above, the elements of a dataset pair profile can be classified into four main groups: statistics, quantitative and qualitative lists, a description model and the types of linking problems exposed (LPTs). We will now describe the processing used for each of these groups.

### 4.1 Processing for the statistics and the quantitative and qualitative lists

Most of our extractions were performed using SPARQL queries encapsulated in JAVA classes. Fig. 10 shows the SPARQL query used to extract from an RDF graph the “OutDegree” statistical value indicating the topic usage ratio by comparing the total number of topics with the number of unique topics, while ensuring that the topic is an IRI.

Fig. 11 shows the SPARQL query used to extract “PropertyUsageDistinctPerSubject” statistical values from an RDF graph, calculating aggregated property usage statistics for each distinct subject.

Similarly, for the creation of a list, Fig. 12 shows the SPARQL query used to extract from an RDF graph the various properties with their usage, as well as the different classes and their usage for each domain.

### 4.2 Processing for the description model

**Fig. 7:**
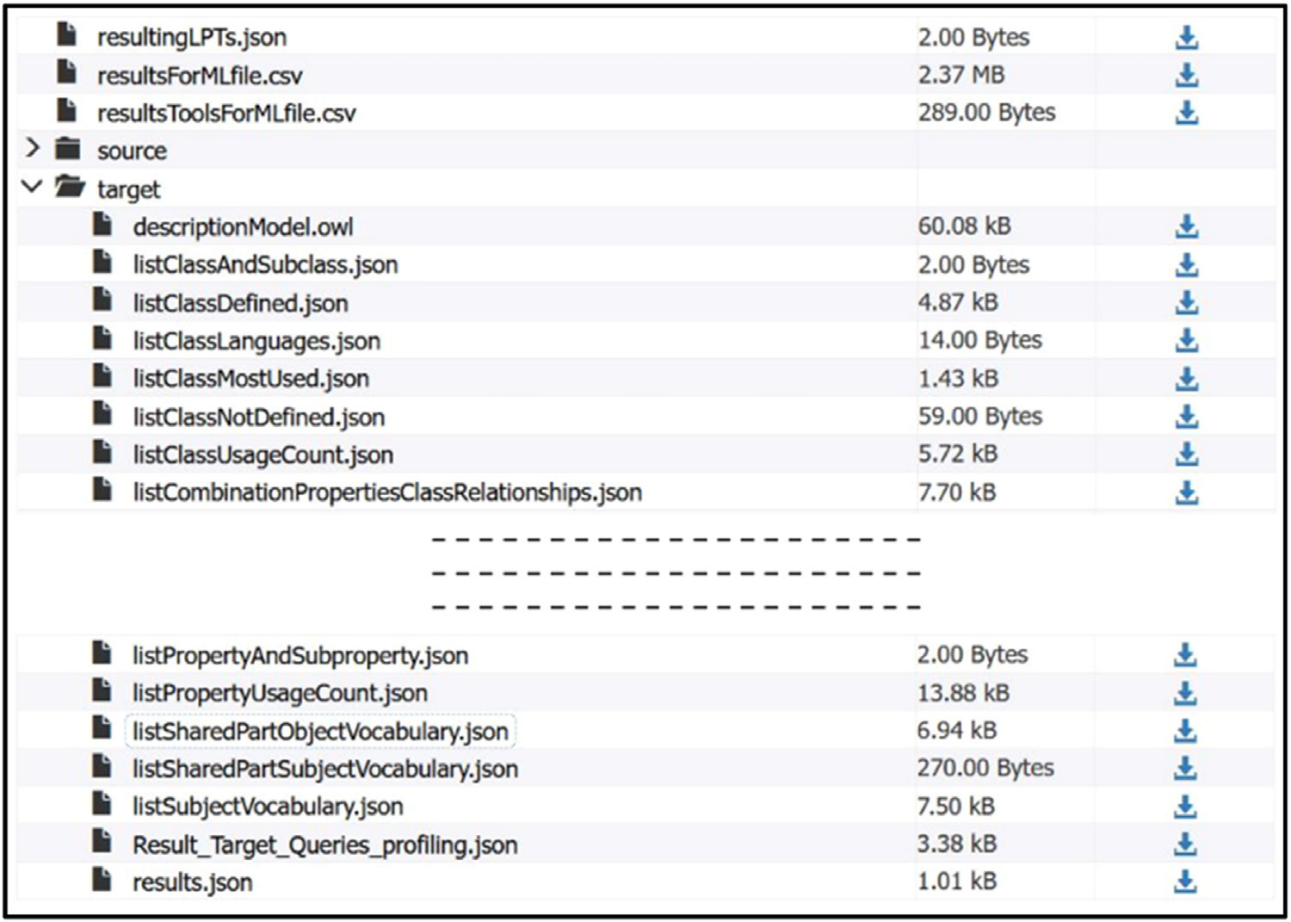
Details of an archive file in the Profiles folder.

For each graph, we present a descriptive model in the form of an OWL ontology contained in the descriptionModel.owl file (see Fig. 7). This model does not claim to be exhaustive, but is based on the most commonly used classes and relations in the graph, providing a structured and coherent view of the data.

**Fig. 8:**
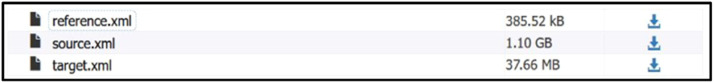
Details of an archive file in the RDF graph pairs folder.

**Fig. 9:**
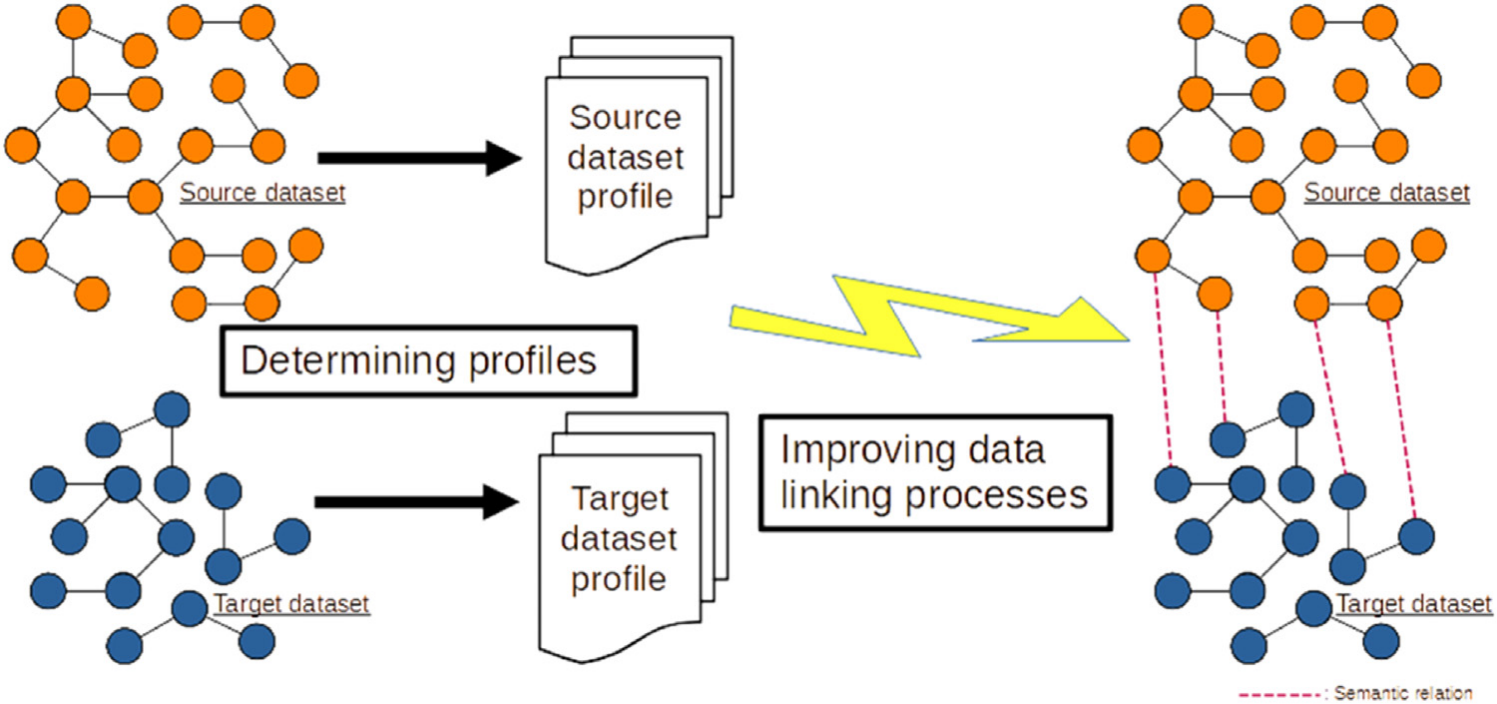
Determining profiles to improve data linking.

**Fig. 10:**
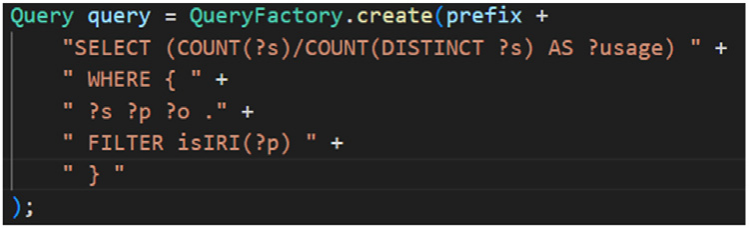
SPARQL query for determining the OutDegree statistical value.

**Fig. 11:**
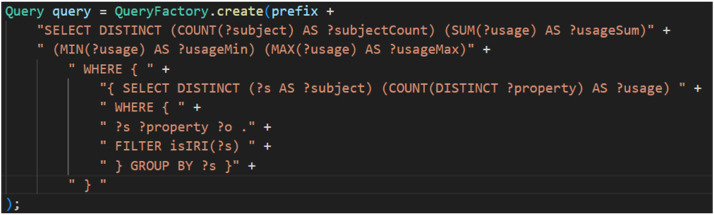
SPARQL query for statistical value determination PropertyUsageDistinctPerSubject.

**Fig. 12:**
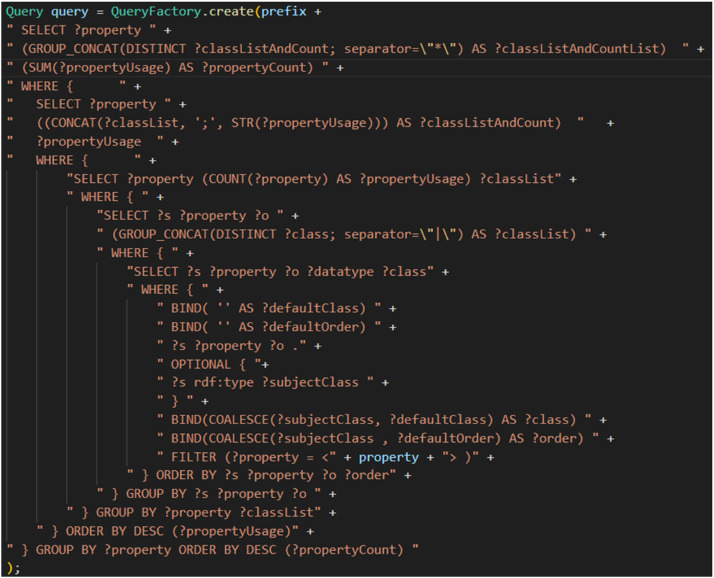
SPARQL query for creating a list of properties with their domain classes and usage.

For the methodology, we used a series of previously generated lists to identify the key elements of our descriptive model. These lists include classes and subclasses, the most frequently used properties, as well as relationships and combinations of properties:

• listClassAndSubclass.json: Provides the class hierarchy.
• listMostUsedObjectProperty.json: Indicates the most common object properties.
• listPropertyUsageCount.json: Shows how often each property is used.
• listCombinationPropertiesPerSubject.json: Shows property combinations for each subject.
• listCombinationPropertiesClassRelationships.json: Describes relationships between classes. These classes can be present in the graph or created for the purpose using the Formal Concept Analysis (FCA) method.
• listMostUsedPropertyWithClassDomain.json: Shows the domain for each of the most used properties.
• listMostUsedPropertyWithDatatypeAndClassRange.json: Shows range for each of the most used properties.

The Formal Concept Analysis method (FCA) [16] is a method for organising and understanding the relationships between objects and their attributes. It can be used to find groups of objects that share the same attributes. When a graph contains only instances (Abox), the principles of the FCA method allow us to create classes based on the common properties of groups of instances.

**Fig. 13:**
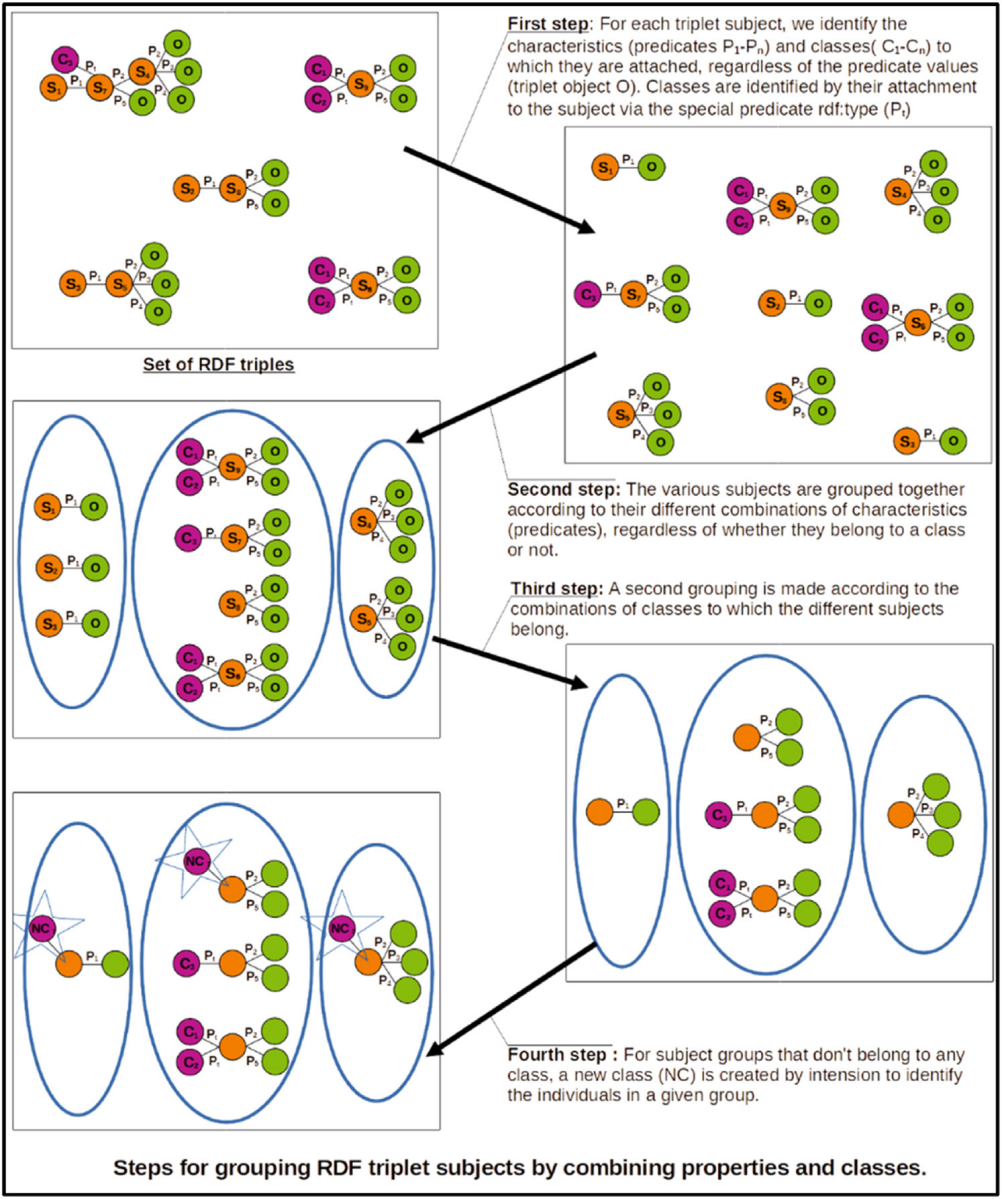
Grouping by combination of properties and classes according to the principles of the FCA method.

Fig. 13 briefly illustrates the operation of our application based on this principle:

• In the first step, we list the properties and classes attached to all the entities (subjects in RDF triple) in the graph.
• In a second step, we group all entities by combination of properties.
• In a third step, we group all entities by combination of properties and classes.
• In a fourth step, we declare groups of entities not attached to a class as instances of a new class created by intension “the class of entities possessing a given property combination”.

This approach makes it possible to define classes by intension (i.e. to define a concept by the set of predicates attached to it), based on combinations of properties common to a set of instances. These intensional classes are crucial for reverse-engineering a model, especially when an explicit model is not provided within the RDF graph.

**Fig. 14:**
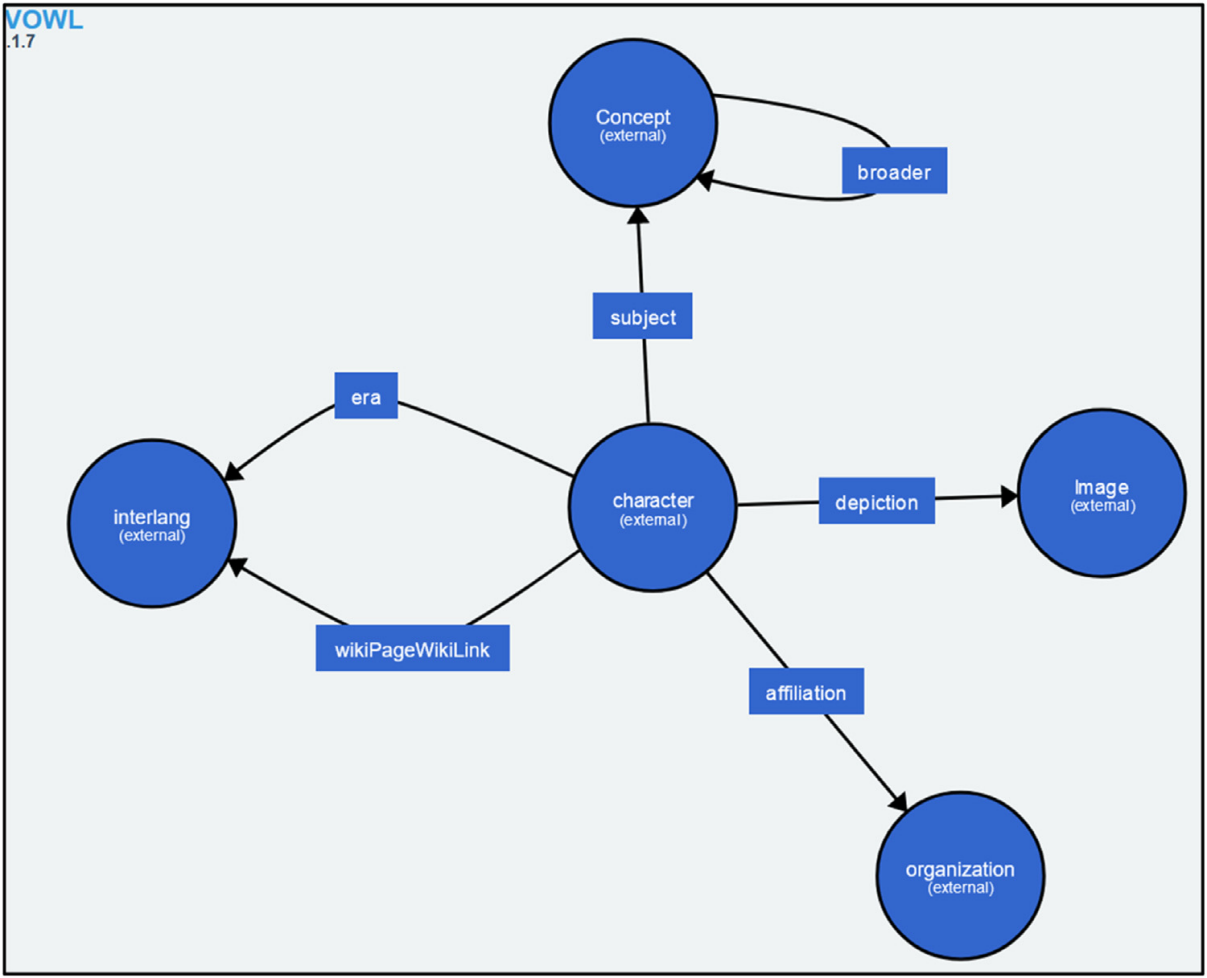
An example of the descriptive model generated by the Dataset Pair Profiling program for the Starwars ontology [15] as seen through the VOWL visualization software.

An example of the descriptive model generated by our program for the starwars ontology [17] is avialable in Fig. 14 via the VOWL visualisation (An online ontology visualization program).

### 4.3 Processing for the linking problem types exposed

As part of the Data-Centric AI-driven Data Linking (DACE-DL) project [8], we are proposing a paradigm shift in data linking by focusing on a bottom-up, data-centric methodology [18]. To fulfil the objectives of this project, we have proposed a classification of different types of linking problems (LPTs) [19] to help the linked data community identify problems in advance and develop more effective solutions.

**Fig. 15:**
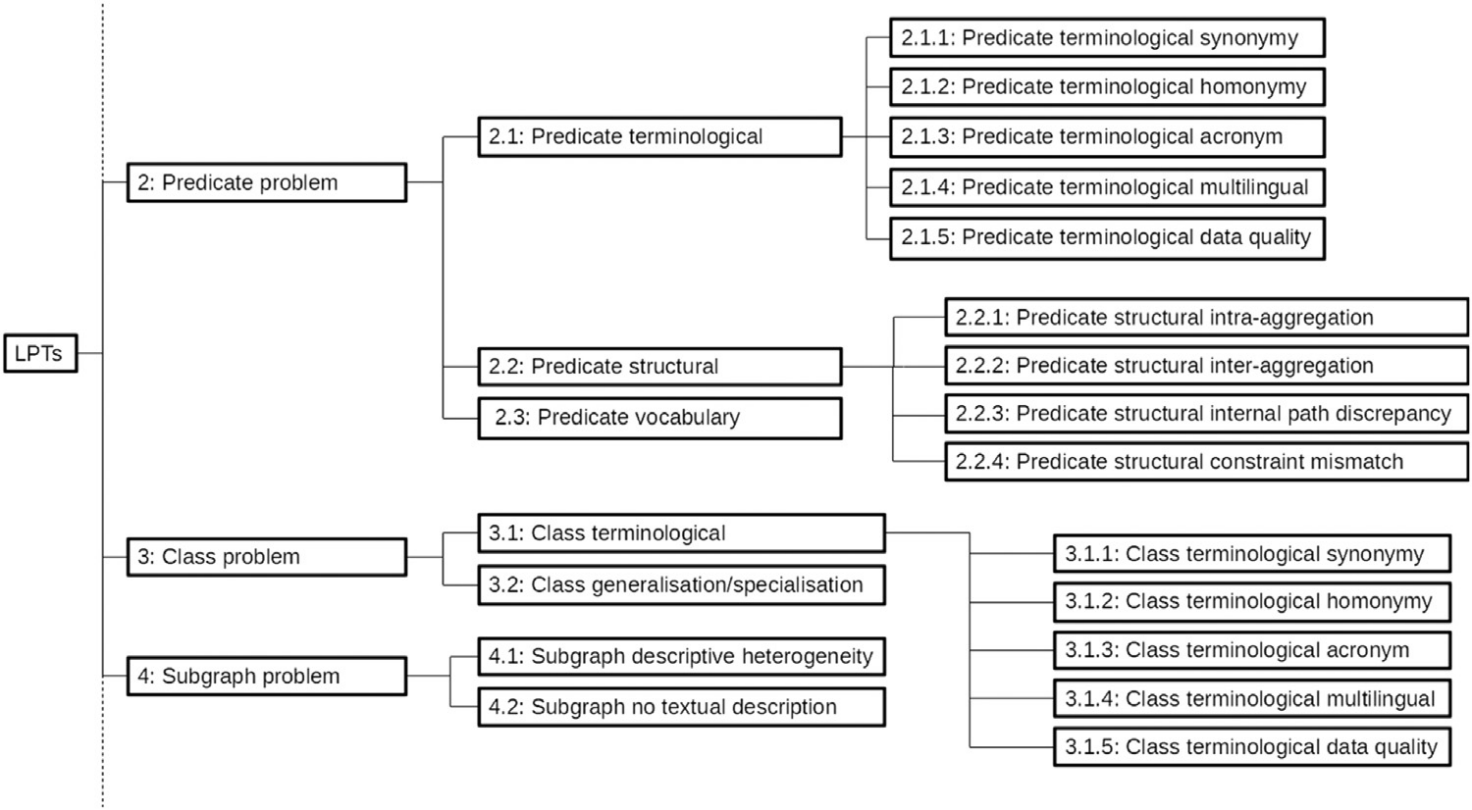
Extract of LPTs classification.

At the first hierarchical level of this classification are five groups of problems that can be encountered during a semantic data linking process: Predicate value problem; Predicate problem; Class problem; Subgraph problem and Graph Problem. An extract from this classification can be seen in Fig. 15.

**Fig. 16:**
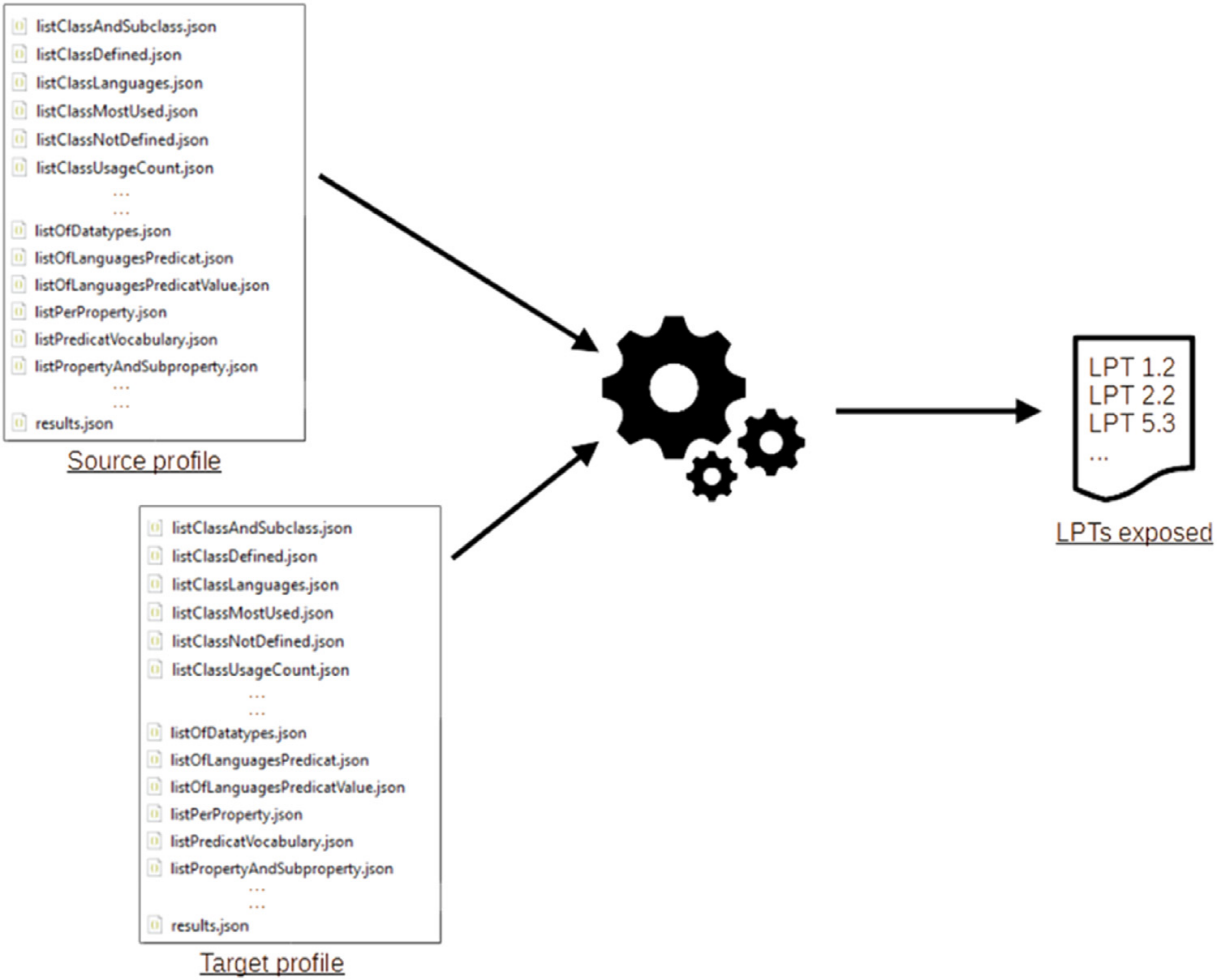
LPTs exposed by a pair of dataset.

Based on the profiles generated for a pair of graphs, a dozen of these LPTs are automatically extracted when they are exposed (see Fig. 16).

The list of potentially exposed LPTs is:

• LPT 1.1.1.2: Predicate value format value type
• LPT 1.1.2.4: Predicate value terminological multilingual problem
• LPT 1.1.3: Predicate value best practice problem
• LPT 2.1.4: Predicate terminological multilingual problem
• LPT 2.1.5: Predicate terminological data quality problem
• LPT 2.2.1: Predicate structural intra-aggregation problem
• LPT 3.1.5: Class terminological data quality
• LPT 3.1.4: Class terminological multilingual problem
• LPT 3.2: Class generalization/specialization problem
• LPT 4.2: Subgraph no textual description problem
• LPT 5.7: Graph scalability Problem
• LPT 5.8: Graph lack of domain ontology Problem

The LPTs exposed for a pair of graphs are logged by the “Dataset Pair Profiling” program in the resultingLPTs.json file (see Fig. 3).

To produce the profiles, we used a laptop personal computer running Windows 10 Professional and equipped with an Intel(R) Core(TM) i7 (2.8GHz) processor and 16GB RAM. The processing time for our largest pair of RDF graphs, TAXREF [13] and the NCBI Taxonomy database [14] (3 million and 15 million triplets respectively), was around an hour and a half.

At the end of the process, the results are stored as JSON files and an OWL file for the description model.

The various results generated by the “Dataset Pair Profiling” program were verified manually by us on the 33 dataset pairs processed, as well as on RDF graphs designed specifically for testing purposes.

This program is publicly available on GitHub [20].For reasons of code clarity and ease of maintenance, the Dataset Pair Profiling program has been developed in a modular fashion, so that each generation of statistics or lists is managed by a single module. Modules can therefore be re-used by different programmers.

## Limitations

Despite the care we have taken in designing our Dataset Pair Profiling program, we are aware that we have no formal means of verifying the results generated. Furthermore, the processing of a larger number of pairs of RDF graphs was also lacking in the validation of our algorithms.

## Ethics Statement

Our current work does not involve human subjects, animal experiments or data collected from social media platforms. The RDF graphs used are public and free to use.

We further confirm that we have read and complied with the ethical requirements for publication in Data in Brief.

## CRediT Author Statement

**Conde Salazar R․:** Conceptualization, Methodology, Software, Investigation, Writing – original draft, Writing – review & editing. **Symeonidou D․:** Supervision, Validation, Writing – review & editing. **Jonquet C․:** Supervision, Validation, Writing – review & editing.

## Data Availability

DataverseProfiles (Original data).
